# Malpositioned Central Line in A Neonate Presenting as Superficial Abdominal Abscess

**DOI:** 10.21699/jns.v6i1.406

**Published:** 2017-01-01

**Authors:** Manasi Garg, Nishanth Rajan, Anjan Dhua, Lalitha Krishnan

**Affiliations:** 1 Department of Pediatrics, Pondicherry Institute of Medical Sciences; 2 Department of Pediatric Surgery, Pondicherry Institute of Medical Sciences

**Dear Sir**

Peripherally inserted central catheters (PICC) have become a routine in intensive care units. Although very effective, they can have life threatening complications like migration, breakage, thrombosis and colonisation [1]. The tip position of PICC lines must always be confirmed. Fluoroscopic placement is ideal, but cannot be done at the bedside and is costly [2]. Single antero-posterior radiograph is the most commonly used and convenient method. Malposition can lead to grave consequences like extravasations and sepsis. Central line migration and extravasation leading to superficial abscess is rare [3,4]. We report a neonate who developed a superficial abdominal collection following surgery due to extravasation through the PICC line.

A 30 week+4 days preterm female baby was born to a multigravida mother with birth weight of 1512g. After initial resuscitation, the baby was put on continuous positive airway pressure for the first 24 hours. Trophic feeds were started on day 2 and gradually increased till baby reached full feeds by day 6. Due to inadequate breast milk, baby’s feeds were complemented with formula. On day 9, she developed features of NEC. Due to the failure of conservative management and subsequent deterioration in the clinical condition, exploratory laparotomy was done. A right hemicolectomy with ileo-transverse anastomosis was performed. On post-operative day 1, a right femoral PICC (Vygon 28 Fr) was inserted through the great saphenous vein in the ankle and the tip position was confirmed by an antero-posterior radiograph (Fig. 1). TPN with intra-lipids infusion was started. On postoperative day 5, the abdomen over the right iliac fossa (just inferior to the laparotomy wound) was noted to be erythematous, tense and distended. Pediatric surgeons reviewed the case and diagnosed a superficial abscess due to surgical site infection. Incision and drainage was done. The fluid evacuated was blood tinged and yellowish white in color. Gram stain and culture of this fluid was negative for any organism. On the sixth postoperative day, continuous oozing of fluid resembling lipid was noted from the incision site. In view of this, malpositioning and extravasation from the PICC line was considered. A lateral x-ray was done to reconfirm its position (Fig. 2). The PICC line was removed and the drainage site completely healed on post operative day 8. She was discharged on day 30 of life when she gained weight adequately on breast feeding and top up feeds.

**Figure:**
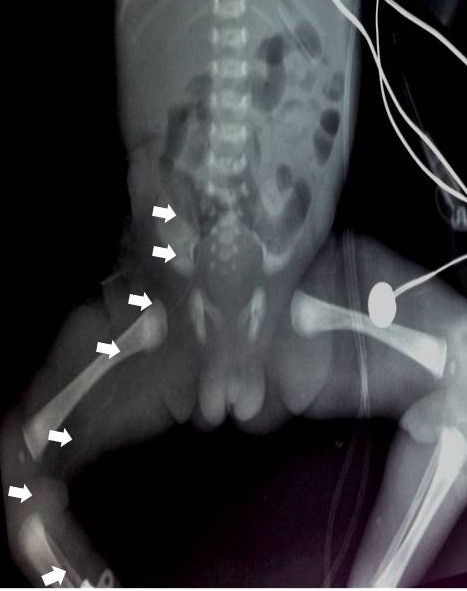
Figure 1: PICC outlined by the arrows.

**Figure:**
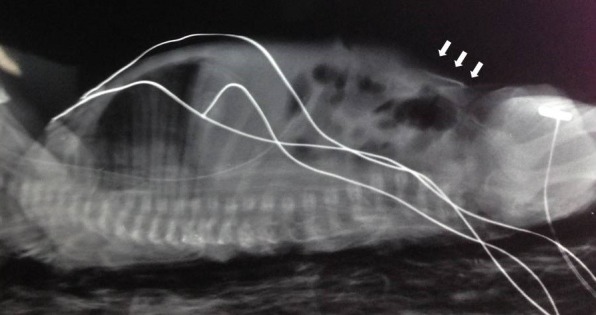
Figure 2: Extravasation of TPN into superficial layer of abdomen

Baker et al. [3] have described extravasation of a lower limb PICC presenting as an abdominal wall abscess like the presentation seen in our patient. Binu et al [4] have described similar case of an upper limb PICC presenting as a chest wall abscess. Confirming catheter tip position of a PICC line is essential to minimise complications. Radiological confirmation with X-rays alone may not be adequate. Fluoroscopic placement was found to be ideal but is costly [2]. Odd, et al. [5] showed that though contrast radiography clearly demonstrates the position of the catheter, precise location of a long line tip may be difficult to determine even with contrast. Coit et al [6] have highlighted the im-portance of two-view radiographs to determine the tip of PICC using the saphenous vein. Katheria et al [7] found that real time ultrasound-guided PICC line insertion by experienced neonatologists was more efficient than standard line placement.

In our case, the initial position of the PICC was thought to be in the internal iliac vein but the tip had found its way to one of the superficial veins of the abdomen. This highlights the fact that all efforts to confirm the tip position should be made before using the PICC line. At the time of insertion, we confirmed the position only with antero-posterior X-ray. Retrospectively, a concomitant lateral X-ray might have helped in detecting the tip in a superficial vein of the abdomen. An attempt was made to locate the tip by bedside ultrasound but it was unsuccessful. Confirmation of catheter tip position and frequent monitoring by experienced personnel will help reducing complications.

## Footnotes

**Source of Support:** Nil

**Conflict of Interest:** None
